# Associations of weight control related behaviors with current cigarette smoking among Chinese adolescents: Results from an ongoing school-based survey in Zhejiang province

**DOI:** 10.18332/tid/192001

**Published:** 2024-09-14

**Authors:** Meng Wang, Jue Xu, Haiping Fang, Liping Yang, Tao Yang, Jianqiang Fan, Xiaofu Du, Chunxiao Xu, Yunqi Guan, Jieming Zhong, Min Yu

**Affiliations:** 1 Zhejiang Provincial Center for Disease Control and Prevention, Hangzhou, China; 2 HangZhou Center for Disease Control and Prevention, Hangzhou, China; 3 Shangyu Center for Disease Control and Prevention, Shaoxing, China; 4 Nanxun Center for Disease Control and Prevention, Huzhou, China; 5 Yuhang Center for Disease Control and Prevention, Hangzhou, China

**Keywords:** adolescents, Chinese, cigarette smoking, health behavior, relationship

## Abstract

**INTRODUCTION:**

Previous studies have suggested that adolescents may smoke cigarettes to control weight, but relevant research is scarce in Chinese youth. This study was conducted to examine the associations of weight control related behaviors with cigarette smoking in Chinese students.

**METHODS:**

This was a secondary analysis of data for 24835 middle and high school students drawn from the 2022 Zhejiang Youth Risk Behavior Survey of China which collected self-reported information of cigarette smoking, weight control strategies and other health-related behaviors. Multivariable logistic regression models were used to estimate the adjusted odds ratios (AORs) with 95% confidence intervals (CIs) for the study associations.

**RESULTS:**

Overall, there were 998 (4.02%) current cigarette smokers in this wave of the Zhejiang YRBS (2022). Neither trying to control weight nor healthy weight control behavior of exercising was associated with current cigarette smoking (AOR=1.15; 95% CI: 0.97–1.37 and AOR=1.01; 95% CI: 0.85–1.20, respectively). Meanwhile, unhealthy weight control behaviors of taking laxatives (AOR=1.52; 95% CI: 1.03–2.26), taking diet pills (AOR=1.82; 95% CI: 1.26–2.62), dieting (AOR=1.24; 95% CI: 1.04–1.49), and fasting (AOR=1.81; 95% CI: 1.40–2.34) were significantly associated with increased odds of current cigarette smoking.

**CONCLUSIONS:**

Screening and developing interventions for unhealthy weight control behaviors should be considered as part of smoking prevention programs among adolescents.

## INTRODUCTION

The tobacco epidemic is one of the biggest public health threats worldwide. In 2020, 22.3% of the world’s population used tobacco and around 80% of the 1.3 billion tobacco users lived in low- and middle-income countries^1^. There is compelling evidence that tobacco smoking is associated with higher risks of morbidity and mortality from a wide range of diseases^2,3^. According to estimations by the Global Burden of Disease Study 2019, smoking tobacco use was accountable for 7.69 million deaths and 200 million disability-adjusted life-years worldwide^4^. With 318 million adult current smokers, China is the largest tobacco consumption country^5^. The extremely high popularity of smoking among Chinese adults calls for more relevant studies on tobacco control strategies.

Notably, adult smoking usually has its roots in adolescence, with most adults who currently use tobacco often started before the age of 21 years^6^. After all, the adolescent brain is particularly susceptible to nicotine addiction and individuals who initiated smoking in adolescence are more likely to be persistent smokers’ later in life^7^. Besides, previous studies also suggest that smoking during adolescence is a strong predictor of adult smoking^8,9^. Thus, to address the tobacco epidemic and reduce the burden of disease caused by tobacco use, more emphasis should be continuously placed on the substantial prevalence of smoking uptake among adolescents and associated factors. According to the most recent Global Youth Tobacco Survey among adolescents aged 13–15 years in 2018, the prevalence of current cigarette smoking was 11.3% in boys and 6.1% in girls^10^. Specific to China in 2019, results from the national survey also showed that the prevalence of self-reported current cigarette use was 14.7%, 8.6%, and 3.9% among students from vocational high school, academic high school, and middle school, respectively^11^. The smoking behavior is prevalent among adolescents and there are many factors behind this phenomenon. Considerable research has explored the smoking related factors among adolescents and findings showed that socioeconomic status, as well as family members or peers smoking, attitudes to smoking, depressive symptoms, and tobacco advertisements and tobacco outlet density were involved^12-14^. Except for these factors that have been previously reported, some studies also proposed that smoking cigarettes might be used as a method of weight control among adolescents^15,16^. In regard to the relationship of weight control attempts and specific behaviors with cigarette smoking among adolescents, current findings in the literature are inconsistent, especially the role of gender, body weight and weight perceptions^17-19^. The aim of the present study is to provide more evidence on the association of weight control related behaviors with current cigarette smoking among Chinese adolescents and to examine whether these associations vary by sex, school type, body mass index (BMI), and weight perceptions.

## METHODS

### Study source

This study was based on a secondary analysis of data from the 2022 Zhejiang Youth Risk Behavior Survey (YRBS) of China, which is an ongoing school-based, cross-sectional study conducted by the Zhejiang Provincial Center for Disease Control and Prevention (CDC). The YRBS study was designed to assess the prevalence of health-related behaviors and associated factors among Chinese students in 2007, and has been conducted every five years since. Details of the study design and sampling strategy have been previously described^20^.

Briefly, this analysis used data from the latest round (the fourth) of Zhejiang YRBS (2022), in which multistage, stratified cluster sampling methods were adopted. Totally, 27070 middle and high school students from 706 classes of 376 schools in 30 counties were recruited, yielding a response rate of 96.53%. Using a self-developed questionnaire taken from the US Youth Risk Behavior Surveillance System (YRBSS) and the Global School-based Student Health Survey (GSHS) as reference, information on relevant health-related behaviors of students was collected in this survey. Without the presence of any teachers, the students filled in the anonymous questionnaire in the classroom by themselves, and then, handed the questionnaires over to the researchers on the spot. To ensure that all the students participated in this survey voluntarily, their parents/guardians and the school officials were sent a written letter and given the option to refuse. Additionally, the confidentiality of the personal data was strictly protected by all the researchers. In particular, the survey abided by the Declaration of Helsinki and was approved by the ethics committee of Zhejiang CDC.

### Assessment of current cigarette smoking

As the main interest of outcome, the current cigarette smoking behavior among adolescents was assessed by the question: ‘During the past 30 days, on how many days did you smoke cigarettes?’ (0; 1–2; 3–5; 6–9; 10– 19; 20–29; or 30 days). Participants were considered current smokers if they answered at least 1 day during the past 30 days.

### Assessment of weight control related behaviors

Weight control related behaviors were the exposure variables of this study. Among these, trying to control weight was assessed by asking: ‘Are you doing something to lose or to keep from gaining weight?’. According to prior knowledge^21-23^, exercising was considered as a healthy weight control behavior, which was assessed by the following question: ‘During the past two years, did you exercise to lose or to keep from gaining weight?’. Unhealthy weight control behaviors that included dieting, taking laxatives, taking diet pills and fasting were assessed with the following questions, respectively: ‘During the past two years, did you eat less food or few calories to lose or to keep from gaining weight?’, ‘During the past two years, did you take laxatives to lose or to keep from gaining weight?’, ‘During the past two years, did you take diet pills without a doctor’s advice to lose or to keep from gaining weight?’, and ‘During the past two years, did you go without eating for 24 hours or more to lose or to keep from gaining weight?’. All the weight control related behaviors were dichotomized with a ‘yes’ or ‘no’ response.

### Other covariates

Sociodemographic characteristics, some lifestyle behaviors and weight perceptions potentially associated with cigarette smoking were simultaneously considered in this analysis. They were age, sex (boys, girls), school location (rural, urban), school type (middle school, academic high school, vocational high school), paternal education level (middle school or below, high school, college or above), maternal education level (middle school or below, high school, college or above), current drinking (yes, no), sleep duration, physical activity (moderate physical activity, and muscle strengthening activity), breakfast intake, fast food intake, BMI (underweight, normal weight, overweight/obese)^24,25^, perceived weight (underweight, normal, overweight), and accuracy of weight perceptions (underestimated, accuracy, overestimated; based on comparisons between self-perceived weight status and actual BMI). Specifically, overestimated perception of weight was underweight adolescents who perceived themselves as being of normal weight or overweight, and normal weight adolescents who perceived themselves as being overweight. Underestimated perception included normal weight adolescents who perceived themselves as being underweight, and overweight/obese adolescents who perceived themselves as being of normal weight or underweight. Overestimated or underestimated weight perceptions were defined as inaccurate. Details of the lifestyle behaviors and weight perceptions are described in Table 1.

**Table 1:** The questionnaire items and response options for assessing lifestyle behaviors and weight perceptions

*Behaviors*	*Items and options*
**Lifestyle**	
**Current drinking**	‘During the past 30 days, on how many days did you have at least one drink of alcohol?’ (0; 1–2; 3–5; 6–9; 10–19; 20–29; or 30 days). Participants who answered at least 1 day during the past 30 days were considered current drinkers.
**Sleep duration**	‘During the past 30 days, on average, how long (hours and minutes) did you sleep every day (including daytime rest)?’. Sleep duration was calculated as: hours + (minutes/60).
**Moderate physical activity**	‘During the past 7 days, how many days did you participate in at least 60 minutes of any kind of physical activity that increased your heart rate and made you breathe harder, such as kicking shuttlecock, fast bicycling, and doing housework, etc.?’ (0;1; 2; 3; 4; 5; 6; or 7 days).
**Muscle strengthening activity**	‘During the past 7days, how many days did you do exercises to strengthen or tone your muscles, such as push-ups, sit-ups, or weight lifting, etc.?’ (0;1; 2; 3; 4; 5; 6; or 7 days).
**Breakfast**	‘During the past 7days, how many days did you eat breakfast?’ (0;1; 2; 3; 4; 5; 6; or 7 days).
**Fast food**	‘During the past 7 days, how many days did you eat fast food, such as hamburger, hotdogs, or potato chips, etc.?’ (0;1; 2; 3; 4; 5; 6; or 7 days).
**Weight perceptions**	
**Perceived weight**	‘How do you describe your weight?’, with response options: very underweight, slightly underweight, about the right weight, slightly overweight, or very overweight. Participants who reported being ‘very’ or ‘slightly’ underweight were categorized as ‘underweight’ and those who reported being ‘very’ or ‘slightly’ overweight were categorized as ‘overweight’.

### Statistical analysis

Among the 27070 middle and high school students recruited in this survey, we excluded those with missing data on age (n=2), sex (n=9), height (n=71) and weight (n=205), or the outliers (below 0.5 percentile or above 99.5 percentile) of height (n=262) and weight (n=262). Besides, in order to keep consistency in BMI cut-offs during the analysis, students aged >18 years (n=1424) were also excluded and finally 24835 adolescents were included.

The prevalence of current cigarette smoking among adolescents with different characteristics was estimated using descriptive statistics and compared using Kruskal-Wallis test and linear-by-linear association chi-squared test. A p<0.05 was considered to be statistically significant. Multivariable logistic regression models were used to estimate the adjusted odds ratios (AORs) with 95% confidence intervals (CIs) for the associations between weight control related behaviors and current cigarette smoking^23^. Adjustments for confounding factors were conducted in four sequential models^26^. In Model 1, sociodemographic characteristics of age, sex, school location, school type, paternal and maternal education level were adjusted. Model 2 was further adjusted for health behaviors of alcohol drinking, physical activity (moderate and muscle strengthening activity), sleep duration, intake of breakfast and fast food, and BMI. Model 3 was adjusted for all variables in Model 2 plus weight perceptions including the self-perceived weight status and the accuracy of weight perceptions. Model 4 was adjusted for all variables in Model 3 plus trying to control weight or specific weight control behaviors. Furthermore, based on Model 4, interaction terms were created and used to test whether the association between weight control related behaviors and current cigarette smoking varied by sex, school type, BMI, self-perceived weight status, and accuracy of weight perceptions. All analyses were performed using SAS statistical package (version 9.4, SAS Institute, Inc., Cary, NC, USA). All statistical tests were based on a two-sided 5% level of significance.

## RESULTS

### Characteristics of study participants

Among the included students (n=24835) with age ranging from 10 to 18 years, there were 998 (4.02%) current cigarette smokers. The basic characteristics of adolescents according to the smoking status are shown in Table 2. The current cigarette users tended to be older (p<0.0001), boys (p<0.0001), from a rural school (p<0.0001), vocational high school (p<0.0001); with lower parental education level (all p<0.0001), current drinking (p<0.0001), longer sleep duration (p<0.0001), higher BMI (p<0.0001); and having a higher frequency of moderate physical activity (p=0.005), muscle strengthening activity (p<0.0001), fast food intake (p<0.0001), a higher proportion of self-perceived underweight status (p<0.0001), underestimated perception of weight (p=0.005), and a lower frequency of breakfast intake (p<0.0001).

**Table 2:** Basic characteristics of Zhejiang school-aged adolescents according to the current smoking status

*Characteristics*	*Current smoking ^a^*		*p ^b^*
	*Yes* *n (%)*	*No* *n (%)*	
**Overall**	998 (4.02)	23805 (95.85)	
**Age** (years), mean ± SD	16.27 ± 1.33	15.57 ± 1.62	<0.0001
**Sex**			<0.0001
Boys	793 (6.23)	11931 (93.77)	
Girls	205 (1.70)	11874 (98.30)	
**School location**			<0.0001
Urban	272 (2.71)	9759 (97.29)	
Rural	726 (4.91)	14046 (95.09)	
**School type**			<0.0001
Middle school	266 (2.16)	12021 (97.84)	
Academic high school	131 (2.01)	6372 (97.99)	
Vocational high school	601 (10.00)	5412 (90.00)	
**Paternal education level**			<0.0001
Middle school or lower	624 (4.59)	12971 (95.41)	
High school	281 (4.25)	6337 (95.75)	
College or higher	92 (2.01)	4496 (97.99)	
**Maternal education level**			<0.0001
Middle school or lower	642 (4.42)	13874 (95.58)	
High school	252 (4.26)	5661 (95.74)	
College or higher	103 (2.36)	4269 (97.64)	
**Current drinking**			<0.0001
No	296 (1.43)	20472 (98.57)	
Yes	697 (17.43)	3301 (82.57)	
**Sleep duration** (hours/day), mean ± SD	8.23 ± 3.40	8.00 ± 1.88	<0.0001
**Moderate physical activity** (days/week), mean ± SD	3.30 ± 2.49	3.08 ± 2.31	0.005
**Muscle strengthening activity** (days/week), mean ± SD	2.46 ± 2.49	2.09 ± 2.36	<0.0001
**Breakfast intake** (days/week), mean ± SD	5.40 ± 2.11	6.14 ± 1.69	<0.0001
**Fast food intake** (days/week), mean ± SD	1.39 ± 1.62	0.65 ± 1.00	<0.0001
**Body mass index** (kg/m^2^), mean ± SD	20.85 ± 3.68	20.33 ± 3.57	<0.0001
**Perceived weight**			<0.0001
Underweight	280 (5.55)	4761 (94.45)	
Normal	328 (3.47)	9114 (96.53)	
Overweight	390 (3.78)	9923 (96.22)	
**Accuracy of weight perceptions**			0.005
Accurate	553 (4.12)	12862 (95.88)	
Underestimated	173 (5.84)	2788 (94.16)	
Overestimated	272 (3.23)	8148 (96.77)	

a The current smoking status data of 32 adolescents were missing.

b For continuous and categorical variables, difference in characteristics of adolescents with or without current smoking behavior was compared using Kruskal-Wallis test and linear-by-linear association chi-squared test, respectively.

### Associations of trying to control weight and weight control behaviors with current cigarette smoking

After adjustment for potential confounders, including sociodemographic characteristics, health behaviors, weight perceptions, and specific weight control behaviors, trying to control weight among adolescents was not significantly associated with the odds of current cigarette smoking (AOR=1.15; 95% CI: 0.97–1.37). Specific to weight control behaviors, the non-significant association with current cigarette smoking was also seen in adolescents with the healthy weight control behavior of exercising (AOR=1.01; 95% CI: 0.85–1.20). By contrast, unhealthy weight control behaviors of taking laxatives (AOR=1.52; 95% CI: 1.03–2.26), taking diet pills (AOR=1.82; 95% CI: 1.26–2.62), dieting (AOR=1.24; 95% CI: 1.04–1.49), and fasting (AOR=1.81; 95% CI: 1.40–2.34) were significantly associated with increased odds of current cigarette smoking (Table 3).

**Table 3:** Adjusted odds ratios of current smoking, by trying to control weight and weight control behaviors, among Zhejiang school-aged adolescents

*Weight control behaviors*	*Cases n/N*	*Current smoking*			
		*Model 1* *AOR (95% CI)*	*Model 2* *AOR (95% CI)*	*Model 3* *AOR (95% CI)*	*Model 4* *AOR (95% CI)*
**Trying to control weight**					
No ®	414/11415	1	1	1	1
Yes	583/13379	1.32 (1.15–1.50)*	1.21 (1.04–1.40)*	1.23 (1.05–1.44)*	1.15 (0.97–1.37)
**Healthy weight control behavior**					
**Exercising**					
No ®	492/12270	1	1	1	1
Yes	486/11738	1.17 (1.02–1.33)*	1.07 (0.92–1.24)	1.09 (0.93–1.28)	1.01 (0.85–1.20)
**Unhealthy weight control behaviors**					
**Taking laxatives**					
No ®	955/24439	1	1	1	1
Yes	43/356	3.17 (2.24–4.49)*	1.59 (1.08–2.35)*	1.58 (1.07–2.34)*	1.52 (1.03–2.26)*
**Taking diet pills**					
No ®	943/24350	1	1	1	1
Yes	53/443	4.41 (3.22–6.04)*	1.85 (1.29–2.66)*	1.86 (1.29–2.68)*	1.82 (1.26–2.62)*
**Dieting**					
No ®	692/18263	1	1	1	1
Yes	304/6530	1.53 (1.33–1.77)*	1.30 (1.10–1.53)*	1.31 (1.10–1.56)*	1.24 (1.04–1.49)*
**Fasting**					
No ®	886/23767	1	1	1	1
Yes	109/1020	4.04 (3.23–5.07)*	1.87 (1.45–2.41)*	1.84 (1.43–2.38)*	1.81 (1.40–2.34)*

AOR: adjusted odds ratio. Model 1: adjusted for age, sex, school location, school type, paternal and maternal education level. Model 2: adjusted as for Model 1 plus health behaviors of alcohol drinking, moderate physical activity, muscle strengthening activity, sleep duration, intake of breakfast and fast food, and body mass index. Model 3: adjusted as for Model 2 plus weight perceptions including self-perceived weight status and the accuracy of weight perceptions. Model 4: adjusted as for Model 3 plus trying to control weight or specific weight control behaviors.

* Significant results. ® Reference categories.

Table 4 shows the Wald chi-squared and p for interactions of sex, school type, BMI, self-perceived weight status, and accuracy of weight perceptions with trying to control weight and weight control behaviors in associations with current cigarette smoking. None of the interaction tests was statistically significant (all p≥0.05), which indicated that these reported associations did not vary statistically by sex, school type, BMI, self-perceived weight status, and accuracy of weight perceptions.

**Table 4:** Wald chi-squared (*χ*^2^) and p-value for interactions of sex, school type, body mass index, self-perceived weight status, and accuracy of weight perceptions, with trying to control weight and weight control behaviors in association with current smoking, among Zhejiang school-aged adolescents

*Interaction term*	*Sex* Wald χ^2^ (p)*	*School type* Wald χ^2^ (p)*	*Body mass index* Wald χ^2^ (p)*	*Self-perceived weight status* Wald χ^2^ (p)*	*Accuracy of weight perceptions* Wald χ^2^ (p)*
Trying to control weight ×	3.67 (0.06)	1.15 (0.28)	1.21 (0.27)	1.51 (0.22)	2.87 (0.09)
Exercising ×	1.41 (0.24)	2.72 (0.10)	1.18 (0.28)	1.59 (0.21)	0.22 (0.64)
Taking laxatives ×	0.54 (0.46)	0.53 (0.46)	0.17 (0.68)	0.004 (0.95)	0.02 (0.89)
Taking diet pills ×	0.27 (0.60)	0.27 (0.60)	0.003 (0.96)	2.93 (0.09)	0.04 (0.84)
Dieting ×	2.86 (0.09)	0.22 (0.64)	1.03 (0.31)	0.20 (0.65)	0.91 (0.34)
Fasting ×	3.40 (0.07)	0.57 (0.45)	3.69 (0.05)	1.41 (0.24)	3.79 (0.05)

* The interaction terms tests were based on Model 4 which adjusted for all the sociodemographic characteristics, health behaviors, and weight perceptions.

## DISCUSSION

With the latest data from the large ongoing school-based survey Zhejiang YRBS (2022) (the fourth round) in Zhejiang Province of China, this study was performed to investigate the associations of weight control related behaviors with current cigarette smoking among Chinese school-based adolescents aged 10–18 years. After adjustment for various potential confounders, analysis results suggested that adolescents who engaged in unhealthy weight control behaviors (i.e. taking laxatives, taking diet pills, dieting, and fasting) were more likely to be current cigarette smokers compared to those who did not engage in these behaviors. However, the significant association with current cigarette use was not replicated in adolescents trying to control weight and engaging in the healthy weight control behavior (i.e. exercising). Besides, we also conducted interaction tests and found that these associations did not vary statistically by sex, school type, BMI, and related weight perceptions. This study is one of the few studies to systematically explore whether weight control attempts and specific weight control behaviors are associated with current cigarette smoking among Chinese school-based adolescents.

Regarding the associations between trying to control weight and cigarette smoking among adolescents, there has been some existing research but with inconsistent findings. Using the baseline data from a three-year, prospective cohort study of 6th and 9th grade students in Hungary, the authors reported that there was no difference in smoking prevalence among students who were motivated to lose or gain weight^27^. Based on the 1999 national Youth Risk Behavior Survey in US high school students, analysis results indicated that trying to lose weight was significantly associated with higher odds of cigarette smoking only among female students, but not the males^17^. Except for the sex factor, previous studies also proposed the possible effects of body weight or weight perceptions on the association of weight loss attempts with cigarette smoking. For example, with a sample of 1132 adolescents aged 12–18 years enrolled in the NHANES study, Strauss et al.^18^ found that there was a two-fold increase in smoking among girls with normal weight who reported trying to lose weight, while the prevalence of smoking was similar among overweight girls who tried to lose weight compared with those who did not^18^. With 944 students from the 2008 National College Health Assessment, findings suggested that individuals who reported being underweight or about normal weight and trying to lose weight were not more likely than their counterparts to smoke cigarettes in the past month^19^. Besides, another US study using data from the 2005 Youth Risk Behavior Survey showed that trying to lose weight was associated with current cigarette use among students before, but not after, adjusting for weight control behaviors, which indicated the association might be also affected by specific weight control behaviors^16^. On the basis of these prior findings, this study has considered these factors in logistics models, and the final results consistently confirmed that trying to control weight was not a significant factor for current cigarette smoking among Chinese adolescents.

Meanwhile, we further explored the independent associations of healthy and unhealthy weight control behaviors with current cigarette smoking among adolescents. After analysis, our results showed that students who exclusively engaged in unhealthy weight control behaviors (i.e. taking laxatives, taking diet pills, dieting, and fasting) were more likely to smoke cigarettes in the past month than their counterparts. In spite of the differences in sample size, study design, population characteristic, and measurements of smoking exposures and weight control behaviors, the present findings were relatively consistent with the results of existing studies that showed unhealthy weight control behaviors were associated with higher odds of health risk behavior of cigarette smoking among adolescents^28-30^. With regard to the positive associations between these unhealthy weight control behaviors and cigarette smoking, the most transparent explanation was that adolescents might smoke cigarettes as a strategy of weight control^15,16^. Besides, as the unhealthy weight control behaviors in our study are the mild or extreme dieting behaviors, the positive associations also might be explained by the possible mechanism that the feeling of food deprivation resulted from weight control behaviors (especially the fasting) would induce the substance use, including the tobacco smoking^31^.

### Strengths and limitations

The strengths of this study include the relatively large sample of school-based students, wide adjustment for potential confounders, and the investigation on the associations of weight control related behaviors with current cigarette smoking among Chinese adolescents. There are some limitations of this study. First, given the cross-sectional nature of study design, causality of the reported associations between weight control behaviors and current cigarette smoking cannot be further inferred. Second, the participants’ information of this survey was collected via self-administered questionnaires and the validity was not checked. According to the findings from the previous validation studies^32,33^, we speculated that the data for this analysis, such as the weight perceptions and sleep duration, were subject to reporting bias and underestimated. Third, as the sample of participants were selected from schools in Zhejiang Province, caution is needed in generalizing these findings to the broader Chinese adolescent populations, as some of them are not in the education system.

## CONCLUSIONS

The present study suggests that unhealthy weight control behaviors such as taking laxatives, taking diet pills, dieting, and fasting are independently associated with cigarette use among Chinese adolescents. These significant findings have some implications for both practice and research. First, school health professionals should realize and incorporate the negative consequences of cigarette smoking into the education on weight control strategies. Second, researchers should promote the screening and develop the early interventions for adolescents who engaged in unhealthy weight control behaviors to prevent smoking. Besides, future research is warranted to provide more explanations on the association of cigarette smoking with unhealthy weight control behaviors among adolescents.

## Data Availability

The data supporting this research are available from the authors on reasonable request.
